# Baricitinib alleviates interstitial lung disease in CIA mice by inhibiting macrophage polarization and increase exosomal miR-126a-3p with anti-fibrotic activity *in vitro*

**DOI:** 10.3389/fphar.2026.1747540

**Published:** 2026-04-01

**Authors:** Xi Liu, Lulu Xu, Xue Zhong, Jie Zhang

**Affiliations:** 1 Department of Geriatrics, Chongqing Medical University, Chongqing, China; 2 Department of Geriatrics, Chongqing General Hospital, Chongqing University, Chongqing, China; 3 Neuro Immune Connections and Repair Lab, Department of Immunology and Infection, Biomedical Research Institute, Hasselt University, Diepenbeek, Belgium; 4 University MS Centrum (UMSC), Biomedical Research Institute, Hasselt University, Diepenbeek, Belgium

**Keywords:** JAK/STAT, macrophage, miR-126a-3p, PI3K/AKT/mTOR, RA-ILD

## Abstract

**Background:**

Rheumatoid arthritis-associated interstitial lung disease (RA-ILD) is a severe condition with an unclear pathogenesis. Here, we investigated the effects of baricitinib on lung fibrosis progression.

**Methods:**

A collagen-induced arthritis (CIA) mouse model was established. Lung tissues were analyzed using Western blotting, immunofluorescence staining, immunohistochemical staining, Masson’s trichrome and hematoxylin and eosin staining. Protein expression was assessed *in vitro* using Western blotting and immunofluorescence staining. The cytokine levels in supernatants were measured using ELISA, and macrophage-derived exosomes were identified using transmission electron microscopy and Western blotting, followed by microRNA sequencing analyses. miR-126a-3p-regulated genes were identified using dual-luciferase reporter assays.

**Results:**

*In vivo*, baricitinib reduced iNOS, CD206 and MerTK levels and collagen deposition in the lungs of CIA mice through the JAK/STAT pathway. *In vitro*, baricitinib downregulated the expression of arginase-1, CD206 and MerTK in macrophages and TGF-β and IL-10 in supernatants. Baricitinib also increased miR-126a-3p expression in macrophage-derived exosomes. miR-126a-3p exerted antifibrotic effects by regulating apoptosis and autophagy via the PI3K/AKT1/mTOR pathway. Silencing JAK1 reduced JAK1 and JAK2 expression.

**Conclusion:**

Baricitinib targets the JAK/STAT signaling pathway in macrophages, thereby exerting dual anti-inflammatory and anti-fibrotic inhibitory effects on CIA-ILD mice. Meanwhile, it increases miR-126a-3p secretion by suppressing M2 polarization, further contributing to its anti-fibrotic activity *in vitro* using NIH3T3 cell.

## 1 Introduction

Rheumatoid arthritis (RA) is an autoimmune disease with an unclear pathogenesis that is characterized by systemic inflammation involving facet joints (Li and Wang, 2023; Tardito et al., 2019). Symptoms of RA include swelling, tenderness, warmth, deformation and stiffness, and extra-articular manifestations, such as rheumatoid nodules, Sicca syndrome, anemia of chronic disease and pulmonary involvement, occur in approximately 40% of patients (Kim et al., 2023; Liu et al., 2022). The lung, which is composed of abundant blood vessels and connective tissues, is the most commonly affected organ (Huang et al., 2023). During RA, 30% of patients develop secondary rheumatoid arthritis-associated interstitial lung disease (RA-ILD), leading to a poorer quality of life and a higher mortality rate than those with RA alone (Kadura and Raghu, 2021; Serrano-Combarro et al., 2025; Tardito et al., 2019; Yamakawa et al., 2021).

Macrophages are innate immune cells that possess certain phagocytic abilities and protect against infection, immune responses, and oncogenesis (Rui et al., 2022). Under different conditions, macrophages can polarize into the M1, M2, and hybrid M1-M2 subtypes (Cutolo et al., 2025). The plasticity of macrophages enables them to polarize into proinflammatory M1 macrophages (induced by IFN-γ, LPS, IL-12, IL-23; iNOS serves as a marker), anti-inflammatory M2c macrophages (induced by glucocorticoids or IL-10; CD206, CD163, Arg-1, and MerTK serve as markers), and profibrotic M2a macrophages (induced by IL-4/13; CD206 and Arg-1 serve as markers) (Cheng et al., 2021; Cutolo et al., 2025). In RA patients, synovial tissue macrophages can be classified into CD206^−^MerTK^−^ (pro-inflammatory) and CD206^+^MerTK^+^ cells (anti-inflammatory and pro-fibrotic) (Cutolo et al., 2025). MerTK-overexpressing macrophages promote TGF-β expression in macrophages and collagen expression in lung fibroblasts (She et al., 2023). Macrophages play a key role in the course of ILD through the Janus kinase-signal transducer and activator of transcription (JAK-STAT) signaling pathway (Huo et al., 2022). This signaling pathway is related to various biological processes, including cell proliferation, differentiation, apoptosis, immune regulation, and macrophage polarization (Huo et al., 2022; Traves et al., 2021). Targeting the JAK/STAT pathway in macrophages may represent a novel therapeutic strategy for treating RA-ILD.

miR-126 exerts effects on various biological processes, including angiogenesis, immune response, inflammation, autophagy, apoptosis, coagulation, viral replication, and tumor growth and metastasis (Guo et al., 2025). Notably, miR-126a-3p regulates the PI3K/AKT/mTOR signaling pathway, which is associated with the occurrence of pulmonary fibrosis (Guo et al., 2025; Pan et al., 2023; Wang et al., 2022).

Baricitinib is a dual JAK1/2 inhibitor (non-selective), with IC_50_ values of 5.9 nM and 5.7 nM, respectively (Gu et al., 2023; Urits et al., 2020). Currently, baricitinib is approved for the treatment of RA, atopic dermatitis, severe alopecia areata, and coronavirus disease 2019 (COVID-19) (Salinas et al., 2022). However, its pulmonary effects in RA-ILD and the underlying mechanisms remain unclear. Therefore, this study aimed to investigate the therapeutic potential of baricitinib for RA-ILD and elucidate its mechanisms.

## 2 Materials and methods

### 2.1 Animals and cells

Thirty SPF male DBA/1 mice (6–8 weeks old; 24 ± 2 g) [QQC No. 202243188; permit No. SCXK (SU) 2021–0013] were obtained from Changzhou Cavens Laboratory Animal Co., Ltd., (Changzhou, China). The mice were acclimatized for 1 week under controlled conditions: a 12-h light/dark cycle, 22 °C ± 2 °C temperature, and free access to food and water. NIH3T3, RAW264.7, and HEK293T cells were purchased from Wuhan Pu Nuosai Biotechnology Co., Ltd., (Wuhan, China).

### 2.2 Drugs and reagents

Bovine type II collagen (bCII, 2 mg/mL; 5 mL; Chondrex, Lynnwood, Washington, United States); complete Freund’s adjuvant (CFA, 10 mL) and incomplete Freund’s adjuvant (IFA, 10 mL; Sigma‒Aldrich, St. Louis, Missouri, United States); baricitinib (5 mg; Adooq Bioscience, Irvine, California, United States); transforming growth factor-beta (TGF-β; 5 mg; MedChemExpress, Irvine California, United States); recombinant murine IL-4 and IL-13 (PeproTech, United States); primary monoclonal antibodies against β-actin (Hua’an Biologicals, Jinan Shandong Province, China), smooth muscle actin (SMA), collagen IV (Col IV), collagen III (Col III), collagen I (Col I), fibronectin (Fn), JAK2, JAK1, STAT1, p-JAK1, p-STAT2, p-STAT4, p-STAT5, iNOS(Affinity, China), F4/80 (Servicebio, China), p-JAK2, p-STAT1, p-STAT3, p-STAT6 (Abcam, Cambridge, Cambridgeshire, United Kingdom), MerTK, STAT2, STAT4, STAT5, STAT6, PI3K, p-PI3K, AKT1, p-AKT1, mTOR, p-mTOR, B-cell lymphoma 2 (Bcl-2), cysteine aspartate-specific protease-3 (caspase-3), cysteine aspartate-specific protease-9 (caspase-9), sequestosome 1 (P62), beclin, CD9, CD63, TSG101, ALIX and Calnexin (HuaBio, Hangzhou, Zhejiang Province, China); goat anti-mouse and goat anti-rabbit secondary antibodies (EarthOx, Millbrae, California, United States); siRNAs targeting JAK1 and JAK2 (si-JAK1 and si-JAK2; RiboBio, Guangzhou, Guangdong Province, China); ELISA kits (4Abio, Suzhou, Jiangsu Province, China); H&E staining kit (Servicebio, G1001, China); Masson’s trichrome kit (Servicebio, G1006, China); AG RNAex Pro Reagent (Accurate Biotechnology, Hunan, China); 2×S6 Universal SYBR qPCR Mix, HyperScript^TM^ III miRNA 1st Strand cDNA Synthesis kit (EnzyArtisan, Shanghai, China); Umibio Exosome Lysis Buffer and Umibio exosome extraction reagent (Umibio Biotechnology, Shanghai, China); BeyoGel™ Plus PAGE precast gels; a bicinchoninic acid assay (BCA) protein assay kit (Solarbio, Beijing, China); 5× protein loading buffer (Beyotime, Shanghai, China); PVDF membranes (0.45 μm, Millipore, IPVH00010, Burlington, MA, United States); and enhanced chemiluminescence (ECL) detection reagent (BioSharp, Hefei, Anhui Province, China) were procured from the indicated suppliers.

### 2.3 Experimental grouping

#### 2.3.1 Cell groups

RAW264.7 cells at passage four were assigned to the following groups: a. cells cultured under normal conditions for 12 h (control); b. cells treated with 60 ng/mL IL-4 and IL-13 for 12 h (based on laboratory optimization) (IL-4/13); and c. cells treated with IL-4/13 (60 ng/mL) and baricitinib (1.0 µM, based on laboratory optimization) for 12 h (baricitinib).

The siRNA transfection experiments involved the following groups: a. RAW264.7 cells cultured under normal conditions for 12 h (control); b. RAW264.7 cells transfected with a nontargeting siRNA, followed by treatment with IL-4/13 (60 ng/mL) for 12 h (NC); c. RAW264.7 cells transfected with si-JAK2, followed by treatment with IL-4/13 (60 ng/mL) for 12 h (si-JAK2); and d. RAW264.7 cells transfected with si-JAK1, followed by treatment with IL-4/13 (60 ng/mL) for 12 h (si-JAK1).

For the coculture experiments, the cells were assigned to the following groups: a. RAW264.7 and NIH3T3 cells cultured separately under normal conditions for 12 h and then cocultured for 12 h (control); b. RAW264.7 cells treated with IL-4/13 (60 ng/mL) for 12 h, washed twice with phosphate-buffered saline (PBS), and then cocultured with NIH3T3 cells for 12 h (IL-4/13); and c. RAW264.7 cells treated with IL-4/13 (60 ng/mL) and baricitinib (1.0 µM) for 12 h, washed twice with PBS, and then cocultured with NIH3T3 cells for 12 h (baricitinib).

For extracellular vesicle (EV) treatment, the cells were classified into the following groups: a. NIH3T3 cells cultured under normal conditions for 72 h (control); b. NIH3T3 cells treated with EVs from RAW264.7 cells stimulated with IL-4/13 (60 ng/mL) for 72 h (EV-neg); and c. NIH3T3 cells treated with EVs from RAW264.7 cells treated with IL-4/13 (60 ng/mL) and baricitinib (1.0 µM) for 72 h (EV-Bari).

For the studies of miR-126a-3p function, the following groups were used: a. NIH3T3 cells cultured under normal conditions for 72 h (control); b. NIH3T3 cells treated with TGF-β (10 µg/mL) for 72 h (TGF-β); and c. NIH3T3 cells treated with TGF-β (10 µg/mL) and miR-126a-3p-containing Exosome (100 ng/mL) for 72 h (Exo-126a-3p).

#### 2.3.2 Mouse groups

**FIGURE 1:**
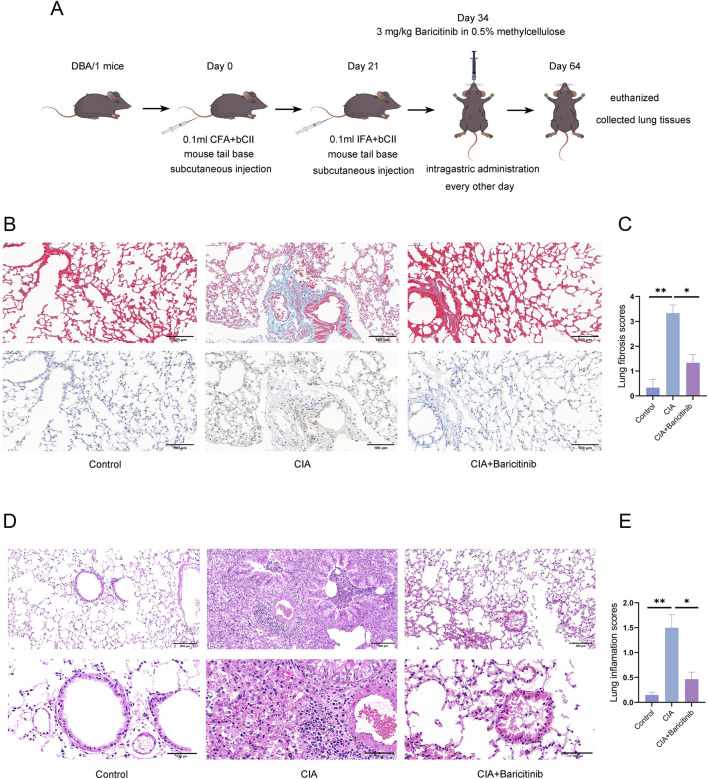
**(A)** Procedures used to establish the CIA mouse model and for the baricitinib intervention. **(B,C)** Masson’s trichrome and F4/80 immunohistochemical staining of mouse lung tissue, and pulmonary fibrosis scoring. **(D,E)** Histological evaluation of mouse lung tissues via hematoxylin and eosin (H&E) staining and subsequent inflammatory scoring.

Nine DBA/1 mice were assigned to the control group and intragastrically administered 0.5% methylcellulose. Twenty-one DBA/1 mice were used to establish a collagen-induced arthritis (CIA) model. On day 0, the mice were injected subcutaneously at the tail base with 0.1 mL of bCII emulsified in CFA. A booster injection of 0.1 mL of bCII in IFA was administered on day 21. Arthritis severity was assessed using a clinical scoring system (0–4 points per paw, with a maximum total score of 16), which started 3 days after the second immunization and was repeated every 3 days. On day 34, the CIA mice were divided into two groups based on the average arthritis score: CIA and CIA+baricitinib groups. The baricitinib-treated group received 3 mg/kg baricitinib in 0.5% methylcellulose via intragastric administration every other day, whereas the CIA group received 0.1 mL of methylcellulose alone (Yaekura et al., 2020). On day 64, all the mice were euthanized by an intraperitoneal injection of 200 mg/kg pentobarbital sodium, and lung tissues were collected for further analysis (Figure 1A). The histopathological analysis revealed that 8 of the 10 lung samples from the CIA group exhibited inflammatory cell infiltration or fibrotic changes; however, only six of the 11 samples from the baricitinib-treated group exhibited similar changes.

### 2.4 Histopathological analysis

#### 2.4.1 Masson’s trichrome staining

Lung tissue samples were fixed in 4% paraformaldehyde for 24 h, embedded in paraffin, and sectioned at 4 μm. After deparaffinization and rehydration, sections were stained with Masson’s trichrome kit according to the manufacturer’s instructions, in which collagen fibers were stained blue, muscle fibers red, and nuclei blue-black (Pan et al., 2023). Fibrosis was graded from 0 to 8 on the Ashcroft scale. Masson’s trichrome-stained sections were scored by dividing them into nine regions and calculating the mean values across these regions. The specific grading criteria were as follows: Grade 0, normal lung tissue; Grade 1, isolated alveolar septa exhibiting mild fibrotic changes; Grade 2, alveolar septa with fibrotic changes and nodular formation; Grade 3, contiguous fibrotic walls within the alveolar septa; Grade 4, single fibrotic mass; Grade 5, confluent fibrotic masses; Grade 6, large, contiguous fibrotic masses; Grade 7, presence of air bubbles; Grade 8, complete fibrous obliteration (Hübner et al., 2008).

#### 2.4.2 Hematoxylin and eosin staining

Lung tissues were fixed with 4% paraformaldehyde for 24 h, dehydrated in graded alcohol solutions, embedded in paraffin, sectioned at 4 µm. Sections were stained with hematoxylin solution for 5 min, differentiated in 1% hydrochloric acid-ethanol for 3–5 s, and rinsed in running water for 15 min (bluing). After staining with eosin solution for 2 min, sections were dehydrated through 95% and 100% ethanol, cleared in xylene, and mounted with neutral balsam. Based on the patterns of lymphocyte infiltration in the perivascular and peritracheal regions, each lung tissue section was divided into nine regions and assessed using a 0–3 point scoring system to calculate the mean value for each region. The scoring criteria were as follows: zero points indicated no infiltration; 1 point indicated occasional lymphocytes; 2 points indicated a thin layer (1–5 cells) of lymphocytes surrounding most bronchi or blood vessels; and three points indicated a thick layer (>5 cells) of infiltrated lymphocytes around most bronchi or blood vessels (Xuan et al., 2024).

### 2.5 Immunofluorescence staining

The tissue sections and cells on slides were blocked for 30 min and incubated with primary antibodies overnight at 4 °C (Shi et al., 2021). Following PBST washes 3 times for 10 min, sections were incubated with FITC-conjugated goat anti-rabbit IgG (Servicebio, 1:500) and Cy3-conjugated goat anti-mouse IgG (Servicebio, 1:500) for 1 h at 37 °C in the dark. Following PBST washes 3 times for 10 min, nuclei were counterstained with DAPI (Servicebio). Sections were mounted with antifade mounting medium (Servicebio) and observed under a confocal laser scanning microscope.

### 2.6 Immunohistochemical staining

Immunohistochemistry was performed on 4-μm paraffin-embedded lung tissue sections. After dewaxing in xylene and rehydration through graded ethanol (100%, 95%, 85%, 75%), antigen retrieval was performed using a pressure cooker with sodium citrate buffer (pH 6.0) at 121 °C for 2–3 min. Endogenous peroxidase activity was blocked with 3% hydrogen peroxide for 15 min at room temperature. Sections were then blocked with 5% bovine serum albumin (BSA) for 30 min and incubated overnight at 4 °C with primary antibodies against F4/80. Subsequently, sections were incubated with horseradish peroxidase (HRP)-conjugated secondary antibody for 1 h at room temperature. Immunoreactivity was visualized using DAB chromogen and counterstained with hematoxylin. Images were captured using a light microscope.

### 2.7 Western blotting

Cells or tissues were lysed in RIPA buffer supplemented with 1% PMSF and phosphatase inhibitor cocktail on ice for 5–10 min; the isolated exosome pellets were resuspended in Exosome Protein Specific Lysis Buffer (UR33101) and incubated on ice for 30 min with intermittent vortexing. After centrifugation at 12,000 × *g* for 15 min at 4 °C, supernatants were collected. Protein concentrations were determined using the BCA assay. Equal amounts of protein (20 μg per lane) were separated by 4%–12% SDS-PAGE and transferred to PVDF membranes via wet transfer. Membranes were blocked for 1 h at room temperature, followed by overnight incubation with primary antibodies at 4 °C. After washing with TBST 3 times for 10 min, membranes were incubated with secondary antibodies for 1 h at 37 °C. Washing with TBST 3 times for 10 min, protein bands were visualized using ECL substrate. Densitometric analysis was performed using ImageJ.

### 2.8 Quantitative real Time-PCR

Total RNA was extracted from lung tissue by Trizol method (Mraz et al., 2009). RNA concentration and purity were assessed by Kaiao K5500Plus MicroSpectrophotometer. Isolated RNA was reversely transcribed using miRNA primers and the HyperScript^TM^ III miRNA 1st Strand cDNA Synthesis kit. Quantitative real-time PCR was performed using 2×S6 Universal SYBR qPCR Mix on a FQD-96A Real-Time PCR System (Bioer, Hangzhou, China). The cycling conditions were: 95 °C for 30 s (initial denaturation), followed by 40 cycles of 95 °C for 10 s and 60 °C for 20 s. U6 was used as the internal reference gene. Relative gene expression was calculated using the 2^^−ΔΔCt^ method. The sequences were as follows:miR-126a-3p-RT: GTC​GTA​TCG​ACT​GCA​GGG​TCC​GAG​GTA​TTC​GCA​GTC​GAT​ACG​ACC​GCA​TT; miR-126a-3p-F: GCG​GCT​CGT​ACC​GTG​AGT​AAT; Mouse-U6-RT: GTC​GTA​TCG​ACT​GCA​GGG​TCC​GAG​GTA​TTC​GCA​GTC​GAT​ACG​ACA​AAA​ATA​T; Mouse-U6-F: AGC​ACA​TAT​ACT​AAA​ATT​GGA​ACG​AT; Common-R: ACT​GCA​GGG​TCC​GAG​GTA​TT.

### 2.9 Dual luciferase assay

Cells were seeded at 30%–50% confluence 1 day before transfection. The plasmid and miRNA were diluted in 100 µL of Opti-MEM, mixed, and incubated at 25 °C for 5 min. The transfection reagents and enhancers were prepared under the same conditions. The two samples were then combined and incubated for 20 min. This mixture was added to the cells and incubated for 4–6 h, after which the medium was replaced with fresh medium. At 48 h posttransfection, the cells were washed with PBS, harvested, and resuspended in 200 µL of medium. A 75-µL aliquot was transferred to a 96-well black-bottom plate, after which 75 µL of firefly luciferase reagent was added. The plate was shaken at 400 rpm for 10 min, and then firefly luciferase activity was measured. Afterward, 75 µL of Renilla luciferase reagent was added, and the activity was recorded under the same conditions.

### 2.10 Exosome enriching and collection

Cell culture supernatants were centrifuged at 300 × *g* for 10 min and 2,000 × *g* for 20 min to remove cells and debris. Exosomes were enrichment using Amicon Ultra-15 centrifugal filter units (100 kDa MWCO, Merck Millipore, UFC9100). Briefly, samples were concentrated by centrifugation at 4,000 × *g* for 30 min at 4 °C to a final volume of 200∼300 μL. The clarified supernatant was mixed with Umibio exosome extraction reagent at a 4:1 ratio (v/v) and incubated at 4 °C overnight. Exosomes were pelleted by centrifugation at 10,000 × *g* for 60 min at 4 °C, resuspended in PBS, and stored at −80 °C for further analysis.

### 2.11 Exosome identification

The exosome morphology was observed using TEM: exosome samples were centrifuged at 10,000 × g, 4 °C for 5 min; add 10 μL of the sample supernatant to the copper grid and let it settle for 2 min, then absorb the supernatant with filter paper; 10 μL of 3% uranyl acetate was added to the copper screen and precipitated for 2 min; the supernatant was removed by filter paper. air drying for several minutes at room temperature; electron microscopy (Hitachi, HT7700) examination performed. Exosomal markers (CD9, CD63, TSG101, ALIX and Calnexin) were detected by WB.

### 2.12 Cell-free miRNA (cf-miRNA) sequencing analysis

High-throughput sequencing of cell-free miRNAs was performed by Cloud-Seq Biotech (Shanghai, China). First, cfRNA was extracted using the GenSeq® cfRNA (cell-free RNA) extraction kit according to the manufacturer’s instructions. Small RNA libraries were constructed using the GenSeq® Small RNA Library Prep Kit (GenSeq, Inc., Shanghai, China) according to the manufacturer’s instructions. Briefly, 3′ and 5′ adaptors were subsequently ligated to RNA samples. The adaptor-ligated RNA was then reverse transcribed into cDNA, followed by PCR amplification. After amplification, the cDNA libraries were size selected for miRNA fractionation, quality control, and quantification using a BioAnalyzer 2100 system (Agilent Technologies, Inc., Santa Clara, California, United States). Sequencing was performed on an Illumina NovaSeq system using the 50-bp single-end mode (Ghosal et al., 2013; Gao et al., 2018; Li and Durbin, 2009; Robinson et al., 2010).

### 2.13 Statistical analysis

Data are presented as mean ± SEM and were analyzed using GraphPad Prism 9.0. Comparisons among multiple groups were performed using either one-way ANOVA followed by Tukey’s HSD test (for equal variances), or Brown-Forsythe ANOVA followed by Games-Howell test (for unequal variances), or Kruskal-Wallis test followed by Dunn’s test (for non-parametric data). P < 0.05 was considered statistically significant.

## 3 Results

### 3.1 Baricitinib reduced lung fibrosis and inflammation in CIA mice

Baricitinib reduced joint swelling in CIA mice (Liu et al., 2023). Lung fibrosis was scored using a standardized histological method (Hübner et al., 2008). Masson’s trichrome staining revealed collagen deposition around the alveoli in the CIA group. This effect was partially attenuated by baricitinib treatment. More specifically, compared with the CIA group, the baricitinib group showed significantly lower collagen deposition, thinner alveolar walls, and fewer fibrotic nodules in the septum. Consistently, immunohistochemistry demonstrated increased macrophage infiltration (F4/80 marker) in the lung interstitium of the CIA group, which was effectively reduced by baricitinib treatment (Figures 1B,C).

HE staining showed that the number of inflammatory cells increased in the blood vessels and around the bronchi of the lung tissue in the CIA group but decreased significantly in the baricitinib group (Figures 1D,E).

### 3.2 Baricitinib suppressed pulmonary M1 and M2 macrophage polarization in CIA mice

**FIGURE 2:**
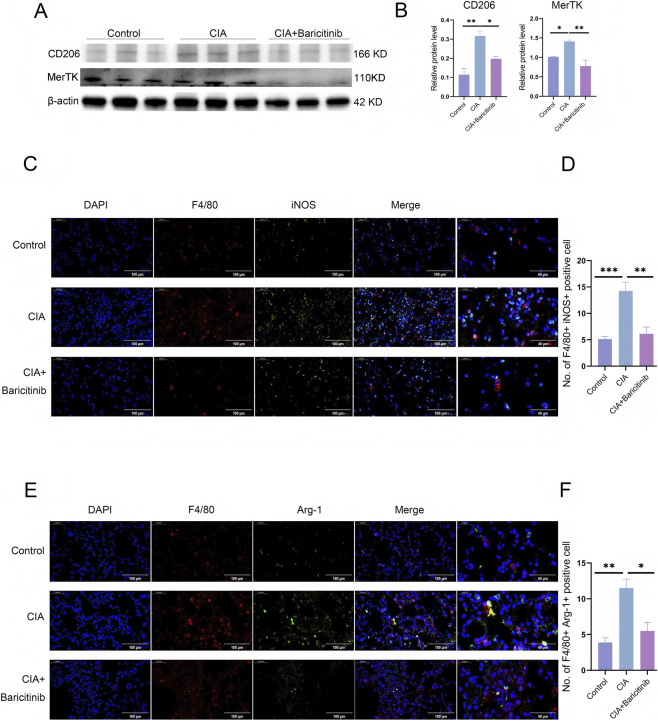
**(A,B)** WB detection of the expression of CD206 and MerTK, markers of M2 macrophages, in mouse lungs. **(C,D)** IF staining showing iNOS expression in mouse lung tissues. **(E,F)** IF staining showing Arg-1 expression in mouse lung tissues. For each lung section, images were captured at ×40 magnification (objective) from five randomly selected fields per mouse, and the number of double-positive cells was quantified using ImageJ.

We performed Western blotting to evaluate whether baricitinib inhibited macrophage polarization in the lungs. We observed that baricitinib treatment reduced the expression of CD206 and MerTK, markers of M2 macrophages (Figures 2A,B). We also performed IF staining to assess M1 and M2 markers in lung tissues. The expression of the macrophage marker F4/80 and the M1 marker iNOS decreased in the baricitinib group (Figures 2C,D). The expression of Arg-1, an M2 marker, was also reduced in baricitinib-treated mice (Figures 2E,F).

### 3.3 Baricitinib suppressed macrophage polarization through JAK1/2–STAT signaling in CIA mice

**FIGURE 3:**
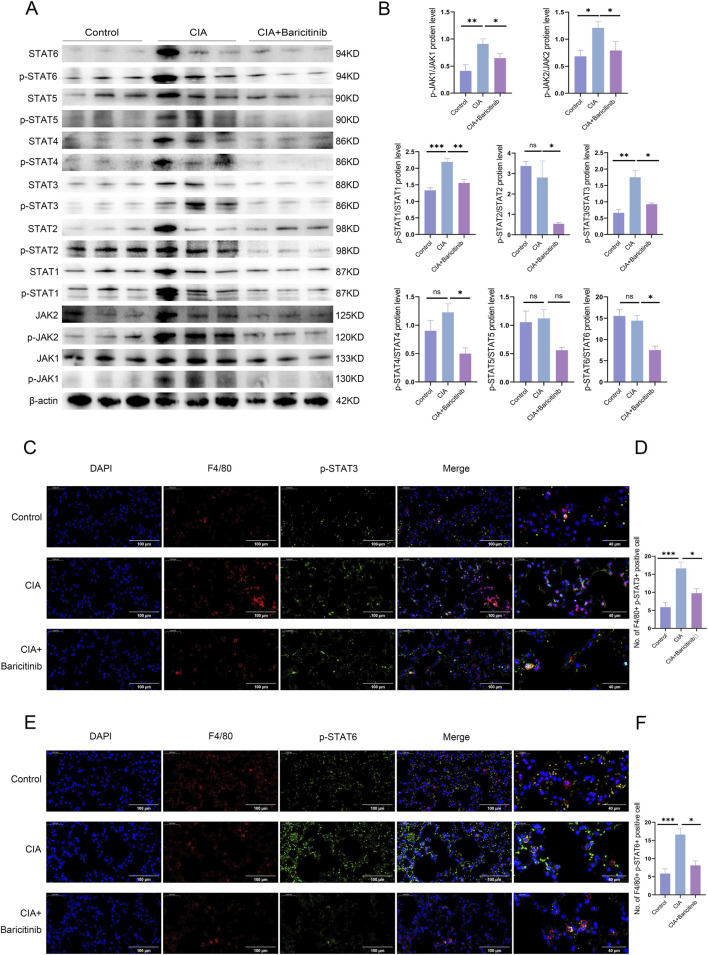
**(A,B)** WB detection of the expression of various proteins involved in the JAK/STAT signaling pathway in mouse lung tissue. **(C–F)** IF staining showing p-STAT3 and p-STAT6 expression in mouse lung tissue. For each lung section, images were captured at ×40 magnification (objective) from five randomly selected fields per mouse, and the number of double-positive cells was quantified using ImageJ.

Western blot analysis of lung tissues revealed that baricitinib treatment reduced the raletive levels of p-JAK1/JAK1 and p-JAK2/JAK2. Downstream signaling molecules, including p-STAT1/STAT1, p-STAT2/STAT2, p-STAT3/STAT3, p-STAT4/STAT4, and p-STAT6/STAT6, were also downregulated (Figures 3A,B). IF staining showed that p-STAT3 levels (Figures 3C,D) and p-STAT6 levels (Figures 3E,F) were increased in the CIA group but were significantly reduced in the baricitinib group.

### 3.4 Baricitinib reduced extracellular matrix (ECM) accumulation through TGF-β, IL-10, and EVs

**FIGURE 4:**
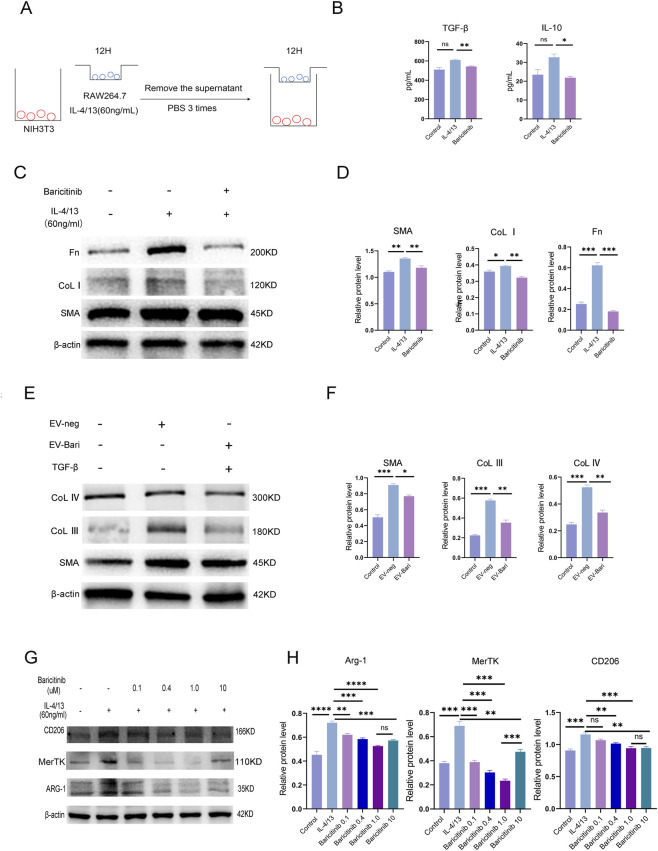
**(A)** Schematic of the coculture model. **(B)** ELISAs for cytokines such as TGF-β and IL-10 in the coculture system. **(C,D)** Expression of Col I, Col III, Fn, and SMA in NIH3T3 cells in the coculture model. **(E,F)** Expression of SMA, Col IV, and Col III in NIH3T3 cells after the macrophage exosome intervention. **(G,H)** CD206,MerTK and Arg-1 expression in RAW264.7 cells after treatment with different concentrations of baricitinib.

In the coculture model (Figure 4A), we measured the levels of cytokines such as TGF-β and IL-10 in the supernatant using ELISAs (Figure 4B). Accordingly, we found that the cytokine levels were significantly lower in the baricitinib group. Additionally, the NIH3T3 cells in the lower layer of the baricitinib-treated cocultures exhibited decreased levels of SMA, Col I, Col III, and Fn (Figures 4C,D). RAW264.7 cells were treated with IL-4/13 in the presence or absence of baricitinib, and EVs were collected from the supernatants and used to treat NIH3T3 cells. Western blot analysis revealed that the levels of SMA, Col IV, and Col III were increased in the EV-neg group (Figures 4E,F), whereas they were significantly reduced in the EV-Bari group.

### 3.5 Baricitinib inhibited the M2 polarization of RAW264.7 cells

IL-4/13 induces the M2 polarization of RAW264.7 cells (Gao et al., 2015). We tested different concentrations of baricitinib (0.1, 0.4, 1.0, and 10 µM) to evaluate their effects on macrophage polarization. CCK-8 assay showed that cell viability was significantly decreased at 10 µM (Supplementary Figure 1). Western blot analysis showed that the expression of CD206, Arg-1 and MerTK was significantly upregulated in the IL-4/13 group (Figures 4G,H). Conversely, this effect was attenuated by baricitinib in a concentration-dependent manner. Compared with 1.0 µM baricitinib, 10 µM baricitinib induced an increase in MerTK expression; however, the expression of CD206 and Arg-1 remained unchanged. Notably, the most pronounced inhibitory effect was observed at a concentration of 1.0 µM baricitinib.

### 3.6 Baricitinib suppressed M2 polarization of RAW264.7 cells through the JAK/STAT pathway

**FIGURE 5:**
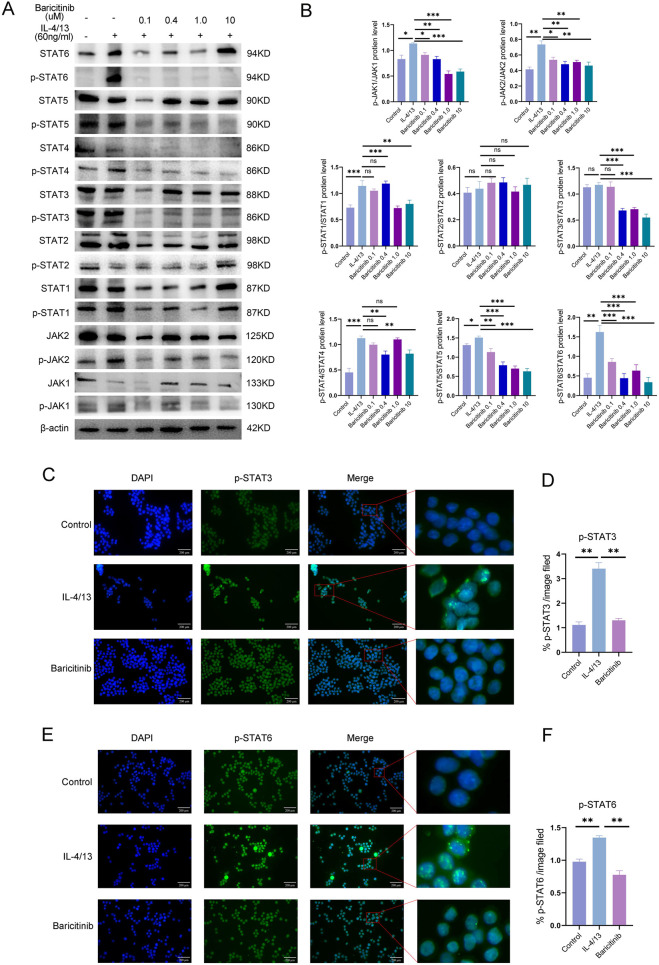
**(A,B)** Changes in the expression of various proteins in the JAK/STAT signaling pathway in RAW264.7 cells treated with different concentrations of baricitinib. **(C-F)** Cellular immunofluorescence staining showing p-STAT3 and p-STAT6 expression in RAW264.7 cells.

Previous studies have shown that baricitinib modulates JAK/STAT signaling in RAW264.7 cells. We tested various concentrations of baricitinib (0.1, 0.4, 1.0, and 10 µM). The raletive levels of p-JAK1/JAK1, p-JAK2/JAK2, p-STAT1/STAT1, p-STAT3/STAT3, p-STAT4/STAT4, p-STAT5/STAT5 and p-STAT6/STAT6 decreased in the baricitinib groups. Moreover, p-STAT2/STAT2 levels showed no significant changes across the study groups (Figures 5A,B). We performed IF staining to confirm the changes in the levels of p-STATs (downstream of JAKs) after baricitinib treatment. The levels of p-STAT3/p-STAT6 were increased in the IL-4/13 group, whereas they were significantly reduced in the baricitinib group (Figures 5C–F). Additionally, the level of p-STAT2 was consistent with the *in vitro* Western blotting results; p-STAT2, p-STAT1 and p-STAT5 remained unchanged levels were observed in all groups (Supplementary Figure 2).

### 3.7 Expression of STATs after silencing JAK1 or JAK2 individually

**FIGURE 6:**
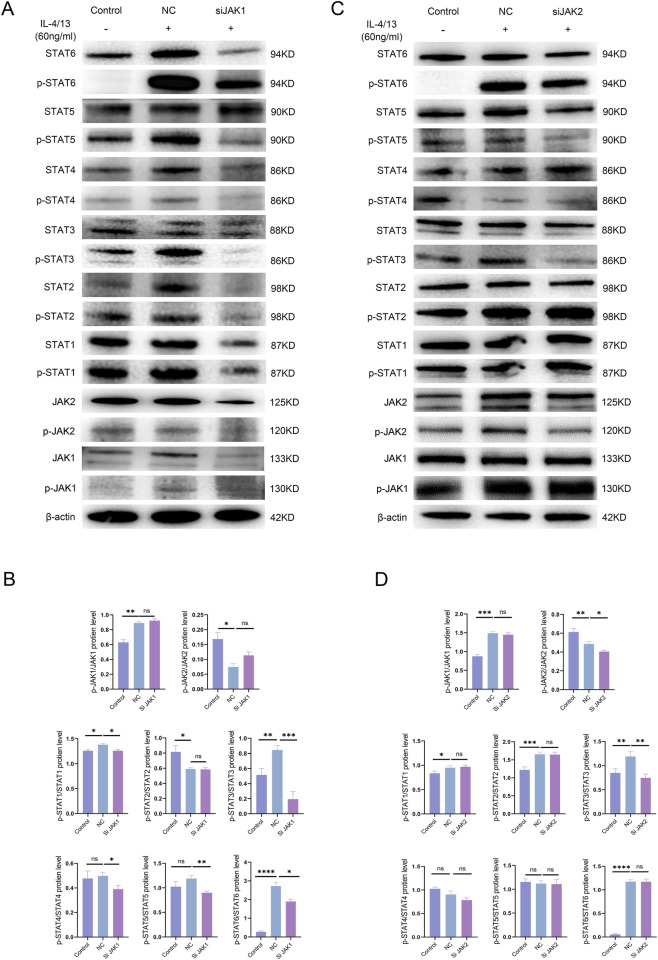
**(A,B)** Changes in the expression of each protein in the JAK/STAT signaling pathway after siRNA-mediated knockdown of JAK1. **(C,D)** Changes in the expression of each protein in the JAK/STAT signaling pathway after siRNA-mediated knockdown of JAK2.

JAK1 knockdown in RAW264.7 cells (siJAK1 group) showed no significant changes the raletive levels of p-JAK1/JAK1 and p-JAK2/JAK2. However, all downstream signaling factors were downregulated except for p-STAT2/STAT2 (Figures 6A,B). JAK2 silencing using an siRNA (siJAK2 group) resulted in reduced p-JAK2/JAK2 and p-STAT3/STAT3 raletive levels (Figures 6C,D).

### 3.8 Exosome identification

**FIGURE 7:**
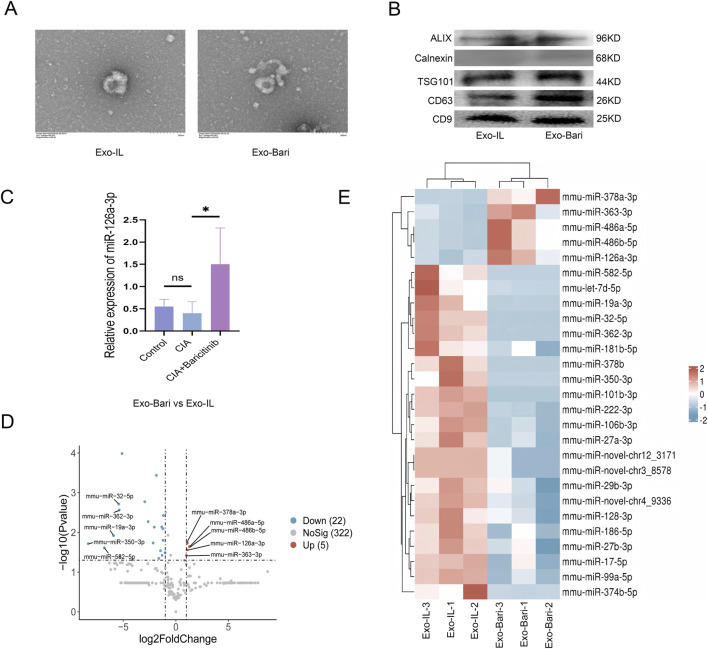
**(A)** Macrophage-derived exosomes were identified using transmission electron microscopy. **(B)** WB detection of the expression of CD9, CD63, TSG101, ALIX and Calnexin, markers of exosome. **(C)** qPCR was used to detect the changes of miR-126a-3p in lung tissue of mice. **(D)** The expression levels of miRNAs in the EV-Bari and EV-IL groups. The blue dots represent downregulated miRNAs in the EV-Bari group, and the red dots represent upregulated miRNAs in the EV-Bari group (fold change >2 and p < 0.05; n = 3). **(E)** Heatmap of the results of the miRNA profiling assays between the EV-Bari and EV-Bari groups (fold change >2 and p < 0.05; n = 3).

TEM images clearly show the vesicular structures characteristic of exosomes (Figure 7A). Western blotting demonstrated positive results for exosome marker proteins (CD9, CD63, TSG101, etc., Figure 7B).

The cf-miRNA sequencing analysis revealed increased miR-126a-3p expression after baricitinib treatment. qPCR demonstrated that the expression of miR-126a-3p was increased in lung tissue in the baricitinib group (Figure 7C).

The cf-miRNA sequencing analysis identified 27 differentially expressed miRNAs (fold change >2.0; p < 0.05), five of which were upregulated in the EV-Bari group compared with the EV-IL group (Figures 7D,E).

### 3.9 miR-126a-3p targets PIK3R2 to regulate PI3K

**FIGURE 8:**
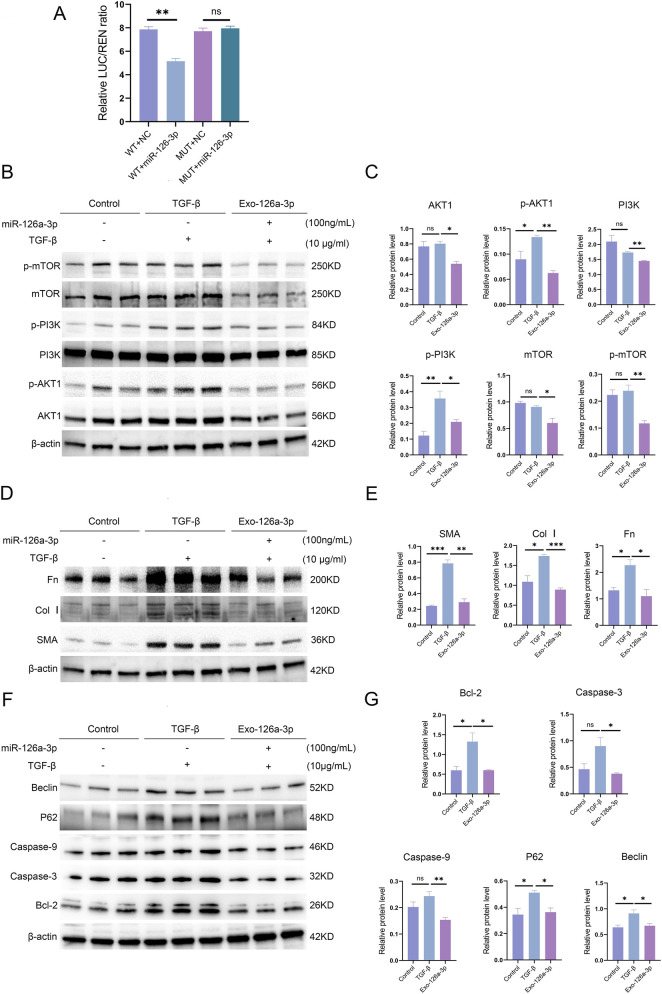
**(A)** The groups and results of the dual-luciferase reporter assay. **(B,C)** Levels of PI3K, p-PI3K, AKT1, p-AKT1, mTOR, and p-mTOR detected in NIH3T3 cells after treatment with TGF-β and miR-126a-3p. **(D,E)** Expression of SMA, Col I, and Fn in NIH3T3 cells after treatment with TGF-β and miR-126a-3p. **(F,G)** Expression of caspase-3, caspase-9, Bcl-2, p62, and beclin-1 in NIH3T3 cells after treatment with TGF-β and miR-126a-3p. *p < 0.05, **p < 0.01, ***p < 0.001, ****p < 0.0001, and ns: not significant.

A dual luciferase assay revealed that miR-126a-3p significantly suppressed luciferase activity in the wild-type PIK3R2 3′UTR (WT) but not in the mutant (MUT; Figure 8A). These results suggested that miR-126a-3p regulates PI3K by directly targeting PIK3R2, potentially influencing related biological processes.

### 3.10 miR-126a-3p inhibits the PI3K/AKT1/mTOR pathway

We used HEK293T cells to produce exosome enriched with miR-126a-3p, which were then applied to NIH3T3 cells along with TGF-β. Western blot analysis showed reduced levels of PI3K/p-PI3K, AKT1/p-AKT1, and mTOR/p-mTOR in the Exo-126a-3p group compared with those in the TGF-β group (Figures 8B,C). Compared with the control group, the TGF-β group showed a significant increase in p-PI3K but no significant difference in total PI3K; there were also no significant changes in total or phosphorylated AKT1 or mTOR.

### 3.11 miR-126a-3p reduces apoptosis, autophagy, and ECM production in NIH3T3 cells

The statistical analysis revealed significant increases in the levels of Col I, Fn, SMA, Bcl-2, P62, and beclin in the TGF-β group compared with those in the control group. However, the expression of Col I, Fn, SMA, Bcl-2, caspase-3/caspase-9, beclin, and P62 was significantly reduced in the Exo-126a-3p group compared with the TGF-β group (Figures 8D–G). The results of miR-126a-3p acting on HFL cells are shown in Supplementary Figure 3.

## 4 Discussion

Macrophages exhibit remarkable plasticity, differentiating into distinct functional phenotypes in response to microenvironmental cues, thereby mediating diverse biological processes (Cutolo et al., 2025; Tardito et al., 2019). Pro-inflammatory macrophages (M1) secrete potent pro-inflammatory cytokines (e.g., TNF-α, IL-1β, IL-6) and various matrix metalloproteinases, promoting extracellular matrix degradation and recruiting additional inflammatory cells to damaged tissues (Huo et al., 2022; Yang et al., 2025). In this study, we observed decreased M1 macrophage markers (iNOS) and reduced vascular/peribronchial inflammatory cells in lung tissue following baricitinib administration, suggesting that baricitinib exerts anti-inflammatory effects by inhibiting M1 polarization. Previous clinical studies have demonstrated that baricitinib improves or maintains pulmonary function and reduces peripheral blood inflammatory markers in RA-ILD patients (d’Alessandro et al., 2020). In RA patients, the mechanism by which the synovial macrophage CD206+MerTK+ cell subset reduces pro-inflammatory cytokine production and promotes the transition from pro-inflammatory to anti-inflammatory macrophage phenotypes may contribute to the alleviation of RA with current treatments (Cutolo et al., 2025). In a study by She et al., elevated MerTK expression was observed in lung tissues of IPF patients and BLM-induced mouse models, and macrophage MerTK was shown to promote fibrogenesis (She et al., 2023). Macrophages also promote fibroblast proliferation via AREG and PDGF and activate fibroblasts through TGF-β, MerTK, and IL-6 signaling, leading to excessive collagen and ECM deposition (Bian et al., 2023; Soldano et al., 2024). Research has shown that depleting both M1 and M2 macrophages mitigates fibrosis and that the M1-to-M2 shift results in anti-inflammatory properties while contributing to fibrosis (Cheng et al., 2021; Cutolo et al., 2021; Campitiello et al., 2024). In this study, we observed reduced levels of MerTK, CD206, and Arg-1 (M2 markers) in lung tissues from the baricitinib group, along with decreased pulmonary collagen deposition, suggesting that baricitinib exerts anti-fibrotic effects by suppressing M2 macrophage polarization. We also observed that the sites of collagen accumulation were accompanied by an increase in macrophages, indicating that macrophage infiltration into the pulmonary interstitium plays a pro-fibrotic role. Collectively, these results demonstrate that baricitinib exerts dual inhibitory effects on inflammation and fibrosis in CIA-ILD mice.

The JAK/STAT pathway is involved in inflammatory and autoimmune diseases, including ILD (Montero et al., 2021). The M1-type macrophages can be activated through the JAK1/JAK2/p-STAT1 pathway to exert pro-inflammatory effects (Yang et al., 2025). JAK1/STAT6 and JAK2/STAT3 promote M2 polarization (Hu et al., 2018; Liu et al., 2024). STAT1 is a key transcription factor involved in fibrosis development (Yuan et al., 2025). *In vivo*, baricitinib, a dual JAK1/2 inhibitor, suppressed macrophage polarization and ameliorated RA-ILD. These findings link JAK/STAT activation in macrophages to RA-ILD progression. In the baricitinib group, the p-STAT1/STAT1, p-STAT2/STAT2, p-STAT3/STAT3, p-STAT4/STAT4 and p-STAT6/STAT6 levels were reduced. Hence, baricitinib exert antifibrotic and antiinflammatory effects by targeting JAK1/2–STAT signaling in macrophages. M2a cells treated with IL-4/IL-13 facilitate the fibroblast-to-myofibroblast transition (FMT) and ECM production *in vitro* (Cheng et al., 2021; Ghebremedhin et al., 2023; Shi et al., 2021). We used a Transwell coculture system with RAW264.7 and NIH3T3 cells to exclude the direct effects of baricitinib and IL-4/13 on fibroblasts. Western blot analysis showed that the supernatants from M2 macrophages promoted collagen and ECM production in NIH3T3 cells. Studies have shown that IL-10 and TGF-β may promote fibrosis (Cheng et al., 2021; Saraiva et al., 2020). ELISA confirmed that the levels of TGF-β and IL-10 were lower in the baricitinib group. These findings indicated that baricitinib inhibits M2 polarization, thereby reducing cytokine secretion and suppressing fibroblast activation, demonstrating its antifibrotic effects.

Recent studies have shown that inhaled LSC-Exos and bronchial epithelial cell-derived EVs can reverse lung fibrosis (Dinh et al., 2020; Kadota et al., 2021). MEx also shifts macrophages toward an anti-inflammatory state (Mansouri et al., 2019). MiRNAs, namely, miR-328, miR-420, miR-7, miR-19a, miR-19b, and miR-26b, have been shown to play an essential role in antifibrotic effects in IPF (Li et al., 2022). In mouse lung tissues, we observed increased levels of miR-126a-3p in the baricitinib group compared to the model group; *in vitro* model sequencing revealed that the expression of miR-126a-3p was significantly upregulated in baricitinib-treated exosome. The miR-126/PIK3R2 pathway reduces myocardial fibrosis, and miR-126a-3p inhibits myoblast migration (Li et al., 2021; Mierzejewski et al., 2023). Furthermore, previous studies have demonstrated that miR-126a-3p can ameliorate fibrosis in post-myocardial infarction and renal models (Jordan et al., 2021). Dual luciferase assays confirmed that miR-126a-3p directly targets PIK3R2 to regulate PI3K/AKT signaling. This pathway is hyperactivated in fibroblasts, macrophages, and epithelial cells during lung fibrosis (Deng et al., 2024; Wang et al., 2022; Zhu et al., 2024). In our study, miR-126a-3p inhibited the PI3K/AKT pathway and reduced ECM production in NIH3T3 cell, indicating that it has antifibrotic effects. Furthermore, extracellular vesicles (EVs) demonstrate superior stability, low immunogenicity, minimal toxicity, and enhanced delivery to and deposition in small airways and alveolar regions (Kadota et al., 2021). Leveraging these advantages, endotracheal administration of EVs containing miR-126a-3p represents a novel therapeutic strategy for RA-ILD patients.

Inadequate autophagy in fibroblasts is a key driver of pulmonary fibrosis, as it: a. promotes extracellular matrix deposition; b. induces fibroblast-to-myofibroblast transformation (FMT), forming a proliferative and apoptosis-resistant phenotype; c. amplifies TGF-β signaling to enhance fibroblast motility (Yue et al., 2022). Both activation of the PI3K/AKT/mTOR pathway and mTOR inhibition (via autophagy dysregulation) contribute to pulmonary fibrosis by conferring anti-apoptotic effects on fibroblasts (Bhatt et al., 2025; Larson-Casey et al., 2016; Wang et al., 2022). We found that exosomes containing miR-126a-3p could downregulate the expression of caspase-3, caspase-9, Bcl-2, Beclin-1, and p62 in NIH3T3 cells. The simultaneous reduction of these proteins appears contradictory to previous findings, which may be attributed to the dual regulatory effects of miR-126a-3p on autophagy and apoptosis (Guo et al., 2025). Alternatively, this discrepancy could stem from limitations of the *in vitro* model, which may not fully recapitulate the *in vivo* microenvironment. However, the precise mechanisms underlying these observations require further investigation.

JAK2 silencing reduced the relative levels of p-JAK2/JAK2 and p-STAT3/STAT3, confirming that p-STAT3/STAT3 is the primary downstream effector of JAK2. JAK1 silencing did not significantly alter the relative levels of p-JAK1/JAK1 or p-JAK2/JAK2, but it reduced the expression of JAK1, p-JAK1, JAK2, and p-JAK2, as well as decreased the levels of all downstream molecules except for p-STAT2/STAT2. Previous studies have shown that JAK1 knockdown reduces JAK2 phosphorylation in hematological malignancies. Coimmunoprecipitation experiments demonstrated that JAK1 is involved in JAK2 phosphorylation in SET-2, WL, and UKE-1 cells (Koppikar et al., 2012). In pulmonary fibrosis, the anti-fibrotic efficacy of selective JAK2 inhibitors may wane over time due to compensatory JAK2 reactivation, which is mediated through upstream JAK1 signaling (Yang et al., 2025). Meanwhile, in rheumatoid arthritis (RA), IFN-γ contributes to disease pathogenesis by activating focal adhesion kinase (FAK) via JAK2-dependent phosphorylation (Kiełbowski et al., 2024). Additionally, TGF-β1 promotes fibrosis by inducing JAK2 phosphorylation, further reinforcing its role in fibrotic signaling cascades (Montero et al., 2021). In summary, we propose that baricitinib, as a JAK1/2 dual inhibitor, may demonstrate reduced pathway escape compared to selective JAK1 or JAK2 inhibitors in alleviating RA-ILD.

## 5 Conclusion

Baricitinib targets the JAK/STAT signaling pathway in macrophages, thereby exerting dual anti-inflammatory and anti-fibrotic inhibitory effects on CIA-ILD mice. Meanwhile, it increases miR-126a-3p secretion by suppressing M2 polarization, further contributing to its anti-fibrotic activity *in vitro* using NIH3T3 cell.

## 6 Limitations

Given that the CIA mouse model exhibits spontaneous resolution of joint swelling after prolonged housing, we cannot exclude the possibility that the observed improvement in lung pathology in the treatment group was due to spontaneous remission; we acknowledge this as a limitation of the study. The lack of pharmacological inhibition assays and reliance on literature-based comparisons have limited the validity of our conclusions. In pulmonary fibrosis, the accumulation of interstitial macrophages is attributed to recruited circulating monocytes, which participate in fibrosis progression (Misharin et al., 2017). Clinical studies have demonstrated that baricitinib effectively reduces fibrosis and inflammation in RA-ILD patients, although its specific immunomodulatory mechanisms remain unclear (d’Alessandro et al., 2020). *In vitro* studies have also shown that MDMs from SSc-ILD patients exhibit enhanced M2 polarization and pro-fibrotic activity compared to normal controls and non-ILD patients (Soldano et al., 2024). A limitation of this study is the lack of direct validation using monocyte-derived macrophages from peripheral blood of RA-ILD patients, necessitating further investigation with clinical samples.

## Data Availability

The data presented in this study are deposited in the NCBI Sequence Read Archive (SRA) repository, accession number PRJNA1367462 (https://www.ncbi.nlm.nih.gov/sra).

## References

[B1] Bhatt J. Ghigo A. Hirsch E. (2025). PI3K/Akt in IPF: untangling fibrosis and charting therapies. Front. Immunol. 16, 1549277. 10.3389/fimmu.2025.1549277 40248697 PMC12004373

[B2] Bian F. Lan Y. W. Zhao S. Deng Z. Shukla S. Acharya A. (2023). Lung endothelial cells regulate pulmonary fibrosis through FOXF1/R-Ras signaling. Nat. Commun. 14, 2560. 10.1038/s41467-023-38177-2 37137915 PMC10156846

[B3] Campitiello R. Soldano S. Gotelli E. Hysa E. Montagna P. Casabella A. (2024). The intervention of macrophages in progressive fibrosis characterizing systemic sclerosis: a systematic review. Autoimmun. Rev. 23, 103637. 10.1016/j.autrev.2024.103637 39255852

[B4] Cheng P. Li S. Chen H. (2021). Macrophages in lung injury, repair, and fibrosis. Cells 10, 436. 10.3390/cells10020436 33670759 PMC7923175

[B5] Cutolo M. Soldano S. Gotelli E. Montagna P. Campitiello R. Paolino S. (2021). CTLA4-Ig treatment induces M1-M2 shift in cultured monocyte-derived macrophages from healthy subjects and rheumatoid arthritis patients. Arthritis Res. Ther. 23, 306. 10.1186/s13075-021-02691-9 34952630 PMC8709961

[B6] Cutolo M. Soldano S. Smith V. Gotelli E. Hysa E. (2025). Dynamic macrophage phenotypes in autoimmune and inflammatory rheumatic diseases. Nat. Rev. Rheumatol. 21, 546–565. 10.1038/s41484-025-01279-w 40721670

[B7] Deng L. Ouyang B. Tang W. Wang N. Yang F. Shi H. (2024). Icariside II modulates pulmonary fibrosis *via* PI3K/Akt/β-catenin pathway inhibition of M2 macrophage program. Phytomedicine 130, 155687. 10.1016/j.phymed.2024.155687 38759312

[B8] Dinh P. C. Paudel D. Brochu H. Popowski K. D. Gracieux M. C. Cores J. (2020). Inhalation of lung spheroid cell secretome and exosomes promotes lung repair in pulmonary fibrosis. Nat. Commun. 11, 1064. 10.1038/s41467-020-14344-7 32111836 PMC7048814

[B9] d’Alessandro M. Perillo F. Metella Refini R. Bergantini L. Bellisai F. Selvi E. (2020). Efficacy of baricitinib in treating rheumatoid arthritis: modulatory effects on fibrotic and inflammatory biomarkers in a real-life setting. Int. Immunopharmacol. 86, 106748. 10.1016/j.intimp.2020.106748 32645631

[B10] Gao S. Zhou J. Liu N. Wang L. Gao Q. Wu Y. (2015). Curcumin induces M2 macrophage polarization by secretion IL-4 and/or IL-13. J. Mol. Cell. Cardiol. 85, 131–139. 10.1016/j.yjmcc.2015.04.025 25944087

[B11] Gao Y. Zhang J. Zhao F. (2018). Circular RNA identification based on multiple seed matching. Brief. Bioinform. 19, 803–810. 10.1093/bib/bbx014 28334140

[B12] Ghebremedhin A. Salam A. B. Adu-Addai B. Noonan S. Stratton R. Ahmed M. S. U. (2023). A novel CD206 targeting peptide inhibits bleomycin-induced pulmonary fibrosis in mice. Cells 12, 1254. 10.3390/cells12091254 37174654 PMC10177262

[B13] Ghosal S. Das S. Sen R. Basak P. Chakrabarti J. (2013). Circ2Traits: a comprehensive database for circular RNA potentially associated with disease and traits. Front. Genet. 4, 283. 10.3389/fgene.2013.00283 24339831 PMC3857533

[B14] Gu S. Liang J. Zhang J. Liu Z. Miao Y. Wei Y. (2023). Baricitinib attenuates bleomycin-induced pulmonary fibrosis in mice by inhibiting TGF-β1 signaling pathway. Molecules 28, 2195. 10.3390/molecules28052195 36903446 PMC10004526

[B15] Guo B. Gu J. Zhuang T. Zhang J. Fan C. Li Y. (2025). MicroRNA-126: from biology to therapeutics. Biomed. Pharmacother. 185, 117953. 10.1016/j.biopha.2025.117953 40036996

[B16] Hu X. Wang H. Han C. Cao X. (2018). Src promotes anti-inflammatory (M2) macrophage generation *via* the IL-4/STAT6 pathway. Cytokine 111, 209–215. 10.1016/j.cyto.2018.08.030 30176559

[B17] Huang H. Chen R. Shao C. Xu Z. Wolters P. J. (2023). Diffuse lung involvement in rheumatoid arthritis: a respiratory physician's perspective. Chin. Med. J. 136, 280–286. 10.1097/CM9.0000000000002577 36689640 PMC10106218

[B18] Hübner R. H. Gitter W. Eddine El Mokhtari N. Mathiak M. Both M. Bolte H. (2008). Standardized quantification of pulmonary fibrosis in histological samples. Biotechniques 44, 507–517. 10.2144/000112729 18476815

[B19] Huo R. Guo Q. Hu J. Li N. Gao R. Mi L. (2022). Therapeutic potential of janus kinase inhibitors for the management of interstitial lung disease. Drug Des. Devel. Ther. 16, 991–998. 10.2147/DDDT.S353494 35400994 PMC8985822

[B20] Jordan N. P. Tingle S. J. Shuttleworth V. G. Cooke K. Redgrave R. E. Singh E. (2021). MiR-126-3p is dynamically regulated in endothelial-to-mesenchymal transition during fibrosis. Int. J. Mol. Sci. 22, 8629. 10.3390/ijms22168629 34445337 PMC8395326

[B21] Kadota T. Fujita Y. Araya J. Watanabe N. Fujimoto S. Kawamoto H. (2021). Human bronchial epithelial cell-derived extracellular vesicle therapy for pulmonary fibrosis *via* inhibition of TGF-β-WNT crosstalk. J. Extracell. Vesicles 10, e12124. 10.1002/jev2.12124 34377373 PMC8329991

[B22] Kadura S. Raghu G. (2021). Rheumatoid arthritis-interstitial lung disease: manifestations and current concepts in pathogenesis and management. Eur. Respir. Rev. 30, 210011. 10.1183/16000617.0011-2021 34168062 PMC9489133

[B23] Kiełbowski K. Plewa P. Bratborska A. W. Bakinowska E. Pawlik A. (2024). JAK inhibitors in rheumatoid arthritis: immunomodulatory properties and clinical efficacy. Int. J. Mol. Sci. 25, 8327. 10.3390/ijms25158327 39125897 PMC11311960

[B24] Kim Y. Yang H. Kim K. (2023). Etiology and pathogenesis of rheumatoid arthritis-interstitial lung disease. Int. J. Mol. Sci. 24, 14509. 10.3390/ijms241914509 37833957 PMC10572849

[B25] Koppikar P. Bhagwat N. Kilpivaara O. Manshouri T. Adli M. Hricik T. (2012). Heterodimeric JAK-STAT activation as a mechanism of persistence to JAK2 inhibitor therapy. Nature 489, 155–159. 10.1038/nature11303 22820254 PMC3991463

[B26] Larson-Casey J. L. Deshane J. S. Ryan A. J. Thannickal V. J. Carter A. B. (2016). Macrophage Akt1 kinase-mediated mitophagy modulates apoptosis resistance and pulmonary fibrosis. Immunity 44, 582–596. 10.1016/j.immuni.2016.01.001 26921108 PMC4794358

[B27] Li H. Durbin R. (2009). Fast and accurate short read alignment with burrows-wheeler transform. Bioinformatics 25, 1754–1760. 10.1093/bioinformatics/btp324 19451168 PMC2705234

[B28] Li Z. Wang X. (2023). Clinical effect and biological mechanism of exercise for rheumatoid arthritis: a mini review. Front. Immunol. 13, 1089621. 10.3389/fimmu.2022.1089621 36685485 PMC9852831

[B29] Li H. Tian X. Ruan Y. Xing J. Meng Z. (2021). Asiatic acid alleviates Ang-II induced cardiac hypertrophy and fibrosis *via* miR-126/PIK3R2 signaling. Nutr. Metab. 18, 71. 10.1186/s12986-021-00596-7 34256802 PMC8278598

[B30] Li S. Zhang J. Feng G. Jiang L. Chen Z. Xin W. (2022). The emerging role of extracellular vesicles from mesenchymal stem cells and macrophages in pulmonary fibrosis: insights into miRNA delivery. Pharmaceuticals 15, 1276. 10.3390/ph15101276 36297388 PMC9610470

[B31] Liu X. Wang Z. Qian H. Tao W. Zhang Y. Hu C. (2022). Natural medicines of targeted rheumatoid arthritis and its action mechanism. Front. Immunol. 13, 945129. 10.3389/fimmu.2022.945129 35979373 PMC9376257

[B32] Liu H. Yang Y. Zhang J. Li X. (2023). Baricitinib improves pulmonary fibrosis in mice with rheumatoid arthritis-associated interstitial lung disease by inhibiting the Jak2/Stat3 signaling pathway. Adv. Rheumatol. 63, 45. 10.1186/s42358-023-00325-z 37641106

[B33] Liu J. Xu L. Guan X. Zhang J. (2024). Experimental study of the effects of pirfenidone and nintedanib on joint inflammation and pulmonary fibrosis in a rheumatoid arthritis-associated interstitial lung disease mouse model. J. Thorac. Dis. 16, 7458–7476. 10.21037/jtd-24-882 39678895 PMC11635228

[B34] Mansouri N. Willis G. R. Fernandez-Gonzalez A. Reis M. Nassiri S. Mitsialis S. A. (2019). Mesenchymal stromal cell exosomes prevent and revert experimental pulmonary fibrosis through modulation of monocyte phenotypes. JCI Insight 4, e128060. 10.1172/jci.insight.128060 31581150 PMC6948760

[B35] Mierzejewski B. Ciemerych M. A. Streminska W. Janczyk-Ilach K. Brzoska E. (2023). miRNA-126a plays important role in myoblast and endothelial cell interaction. Sci. Rep. 13, 15046. 10.1038/s41598-023-41626-z 37699959 PMC10497517

[B36] Misharin A. V. Morales-Nebreda L. Reyfman P. A. Cuda C. M. Walter J. M. McQuattie-Pimentel A. C. (2017). Monocyte-derived alveolar macrophages drive lung fibrosis and persist in the lung over the life span. J. Exp. Med. 214, 2387–2404. 10.1084/jem.20162152 28694385 PMC5551573

[B37] Montero P. Milara J. Roger I. Cortijo J. (2021). Role of JAK/STAT in interstitial lung diseases; molecular and cellular mechanisms. Int. J. Mol. Sci. 22, 6211. 10.3390/ijms22126211 34207510 PMC8226626

[B38] Mraz M. Malinova K. Mayer J. Pospisilova S. (2009). MicroRNA isolation and stability in stored RNA samples. Biochem. Biophys. Res. Commun. 390, 1–4. 10.1016/j.bbrc.2009.09.061 19769940

[B39] Pan L. Cheng Y. Yang W. Wu X. Zhu H. Hu M. (2023). Nintedanib ameliorates bleomycin-induced pulmonary fibrosis, inflammation, apoptosis, and oxidative stress by modulating PI3K/Akt/mTOR pathway in mice. Inflammation 46, 1531–1542. 10.1007/s10753-023-01825-2 37160579 PMC10359208

[B40] Robinson M. D. McCarthy D. J. Smyth G. K. (2010). edgeR: a bioconductor package for differential expression analysis of digital gene expression data. Bioinformatics 26, 139–140. 10.1093/bioinformatics/btp616 19910308 PMC2796818

[B41] Rui Y. Han X. Jiang A. Hu J. Li M. Liu B. (2022). Eucalyptol prevents bleomycin-induced pulmonary fibrosis and M2 macrophage polarization. Eur. J. Pharmacol. 931, 175184. 10.1016/j.ejphar.2022.175184 35964659

[B42] Salinas C. A. Louder A. Polinski J. Zhang T. C. Bower H. Phillips S. (2022). Evaluation of VTE, MACE, and serious infections among patients with RA treated with baricitinib compared to TNFi: a multi-database study of patients in routine care using disease registries and claims databases. Rheumatol. Ther. 9, 201–223. 10.1007/s40744-022-00505-1 36371760 PMC9660195

[B43] Saraiva M. Vieira P. O’Garra A. (2020). Biology and therapeutic potential of interleukin-10. J. Exp. Med. 217, e20190418. 10.1084/jem.20190418 31611251 PMC7037253

[B44] Serrano-Combarro A. Atienza-Mateo B. Martín-Gutiérrez A. Loarce-Martos J. Dubuc C. A. E. Pastor Mena M. (2025). Baricitinib in rheumatoid arthritis-interstitial lung disease: a literature review and national multicentre study of 72 patients. Rheumatology 64, 5471–5480. 10.1093/rheumatology/keaf314 40493905

[B45] She Y. Xu X. Yu Q. Yang X. He J. Tang X. X. (2023). Elevated expression of macrophage MERTK exhibits profibrotic effects and results in defective regulation of efferocytosis function in pulmonary fibrosis. Respir. Res. 24, 118. 10.1186/s12931-023-02424-3 37120511 PMC10148433

[B46] Shi L. Ren J. Li J. Wang D. Wang Y. Qin T. (2021). Extracellular vesicles derived from umbilical cord mesenchymal stromal cells alleviate pulmonary fibrosis by means of transforming growth factor-β signaling inhibition. Stem Cell Res. Ther. 12, 230. 10.1186/s13287-021-02296-8 33845892 PMC8041243

[B47] Soldano S. Smith V. Montagna P. Gotelli E. Campitiello R. Pizzorni C. (2024). Nintedanib downregulates the profibrotic M2 phenotype in cultured monocyte-derived macrophages obtained from systemic sclerosis patients affected by interstitial lung disease. Arthritis Res. Ther. 26, 74. 10.1186/s13075-024-03308-7 38509595 PMC10953168

[B48] Tardito S. Martinelli G. Soldano S. Paolino S. Pacini G. Patane M. (2019). Macrophage M1/M2 polarization and rheumatoid arthritis: a systematic review. Autoimmun. Rev. 18, 102397. 10.1016/j.autrev.2019.102397 31520798

[B49] Traves P. G. Murray B. Campigotto F. Galien R. Meng A. Di Paolo J. A. (2021). JAK selectivity and the implications for clinical inhibition of pharmacodynamic cytokine signalling by filgotinib, upadacitinib, tofacitinib and baricitinib. Ann. Rheum. Dis. 80, 865–875. 10.1136/annrheumdis-2020-219012 33741556 PMC8237188

[B50] Urits I. Israel J. Hakobyan H. Yusin G. Lassiter G. Fackler N. (2020). Baricitinib for the treatment of rheumatoid arthritis. Rheumatology 58, 407–415. 10.5114/reum.2020.102006 33456084 PMC7792534

[B51] Wang J. Hu K. Cai X. Yang B. He Q. Wang J. (2022). Targeting PI3K/AKT signaling for treatment of idiopathic pulmonary fibrosis. Acta Pharm. Sin. B. 12, 18–32. 10.1016/j.apsb.2021.07.023 35127370 PMC8799876

[B52] Xuan W. Wang S. Alarcon-Calderon A. Bagwell M. S. Para R. Wang F. (2024). Nebulized platelet-derived extracellular vesicles attenuate chronic cigarette smoke-induced Murine emphysema. Transl. Res. 269, 76–93. 10.1016/j.trsl.2024.02.001 38325750

[B53] Yaekura A. Yoshida K. Morii K. Oketani Y. Okumura I. Kaneshiro K. (2020). Chronotherapy targeting cytokine secretion attenuates collagen-induced arthritis in mice. Int. Immunopharmacol. 84, 106549. 10.1016/j.intimp.2020.106549 32416449

[B54] Yamakawa H. Ogura T. Kameda H. Kishaba T. Iwasawa T. Takemura T. (2021). Decision-making strategy for the treatment of rheumatoid arthritis-associated interstitial lung disease (RA-ILD). J. Clin. Med. 10, 3806. 10.3390/jcm10173806 34501253 PMC8432201

[B55] Yang Z. Li Z. Liu Z. Li W. Jiao R. Liu Y. (2025). Ruxolitinib attenuates bleomycin-induced pulmonary fibrosis in mice by modulating macrophage polarization through the JAK/STAT signaling pathway. Int. Immunopharmacol. 161, 114962. 10.1016/j.intimp.2025.114962 40466612

[B56] Yuan Z. Lv G. Liu X. Xiao Y. Tan Y. Zhu Y. (2025). Machine learning selection of basement membrane-associated genes and development of a predictive model for kidney fibrosis. Sci. Rep. 15, 6567. 10.1038/s41598-025-89733-3 39994219 PMC11850825

[B57] Yue Y. L. Zhang M. Y. Liu J. Y. Fang L. J. Qu Y. Q. (2022). The role of autophagy in idiopathic pulmonary fibrosis: from mechanisms to therapies. Ther. Adv. Respir. Dis. 16, 17534666221140972. 10.1177/17534666221140972 36468453 PMC9726854

[B58] Zhu J. Jiang Q. Gao S. Xia Q. Zhang H. Liu B. (2024). IL20Rb aggravates pulmonary fibrosis through enhancing bone marrow derived profibrotic macrophage activation. Pharmacol. Res. 203, 107178. 10.1016/j.phrs.2024.107178 38583686

