# Supplementary figures and images for: Baricitinib alleviates interstitial lung disease in CIA mice by inhibiting macrophage polarization and increase exosomal miR-126a-3p with anti-fibrotic activity in vitro
Source: Front Pharmacol. 2026 Apr 1;17:1747540. doi: 10.3389/fphar.2026.1747540 (PMC13079288; doi:10.3389/fphar.2026.1747540)

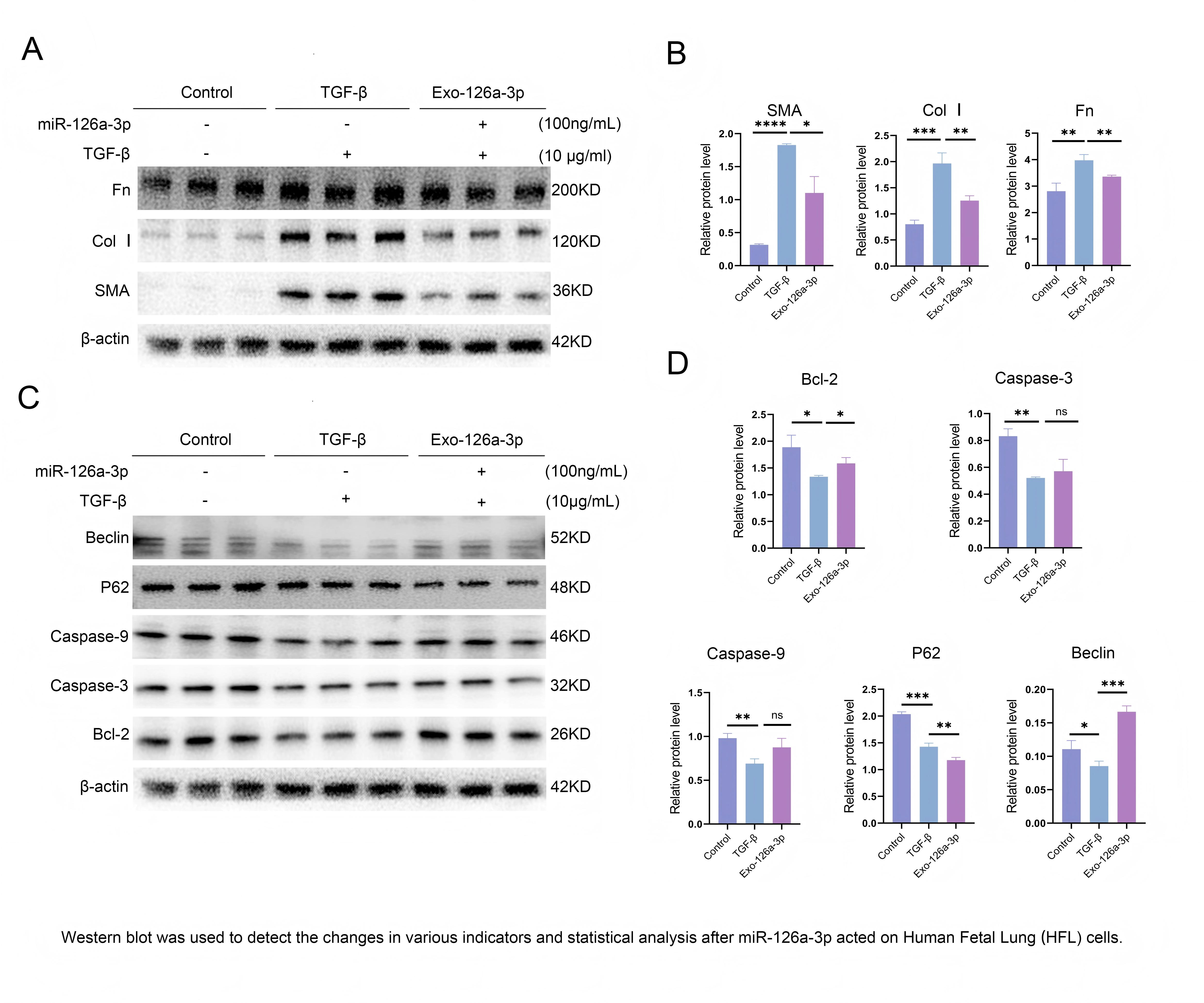

Supplement: Supplementary file 1 [file Image3.jpg]

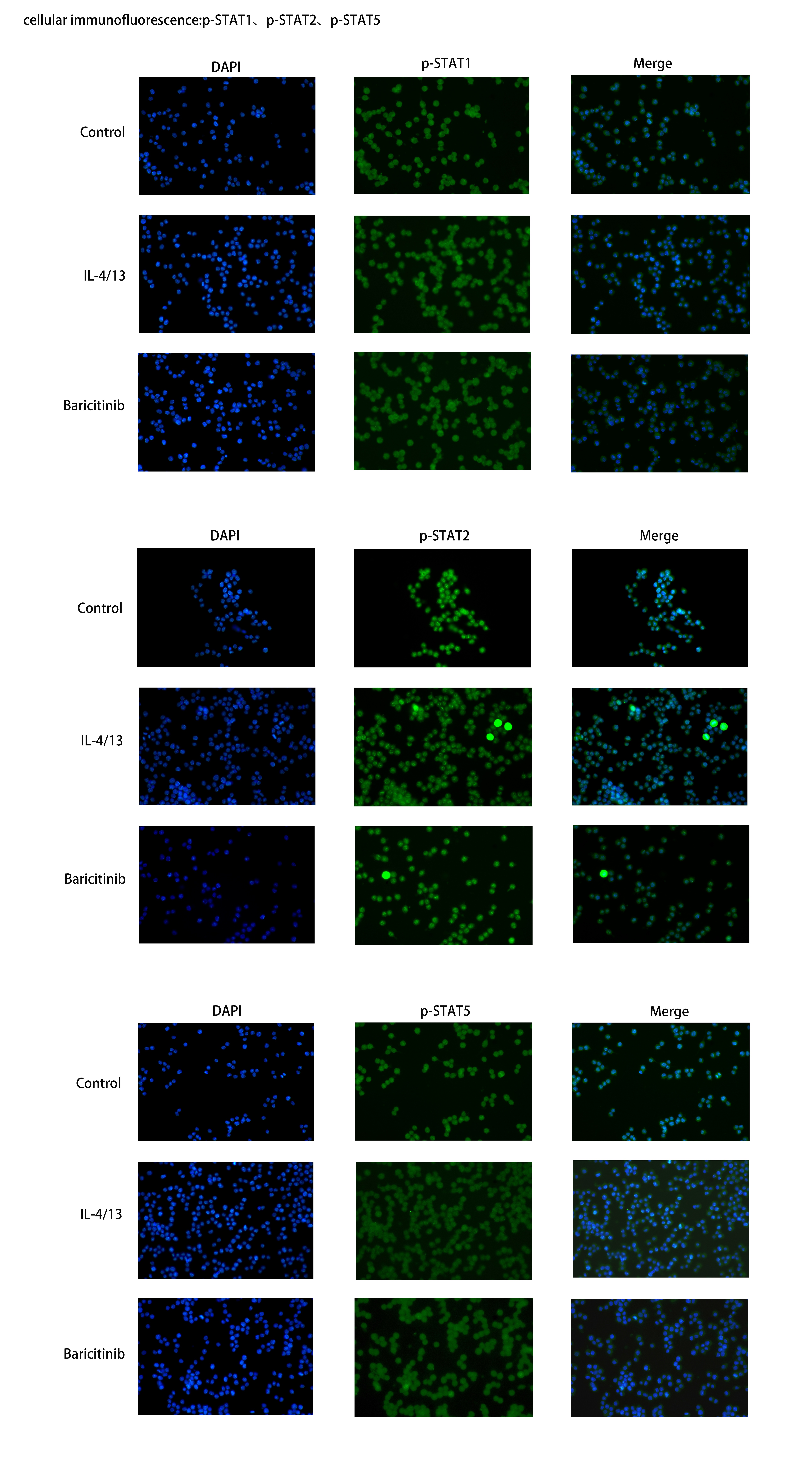

Supplement: Supplementary file 2 [file Image2.png]

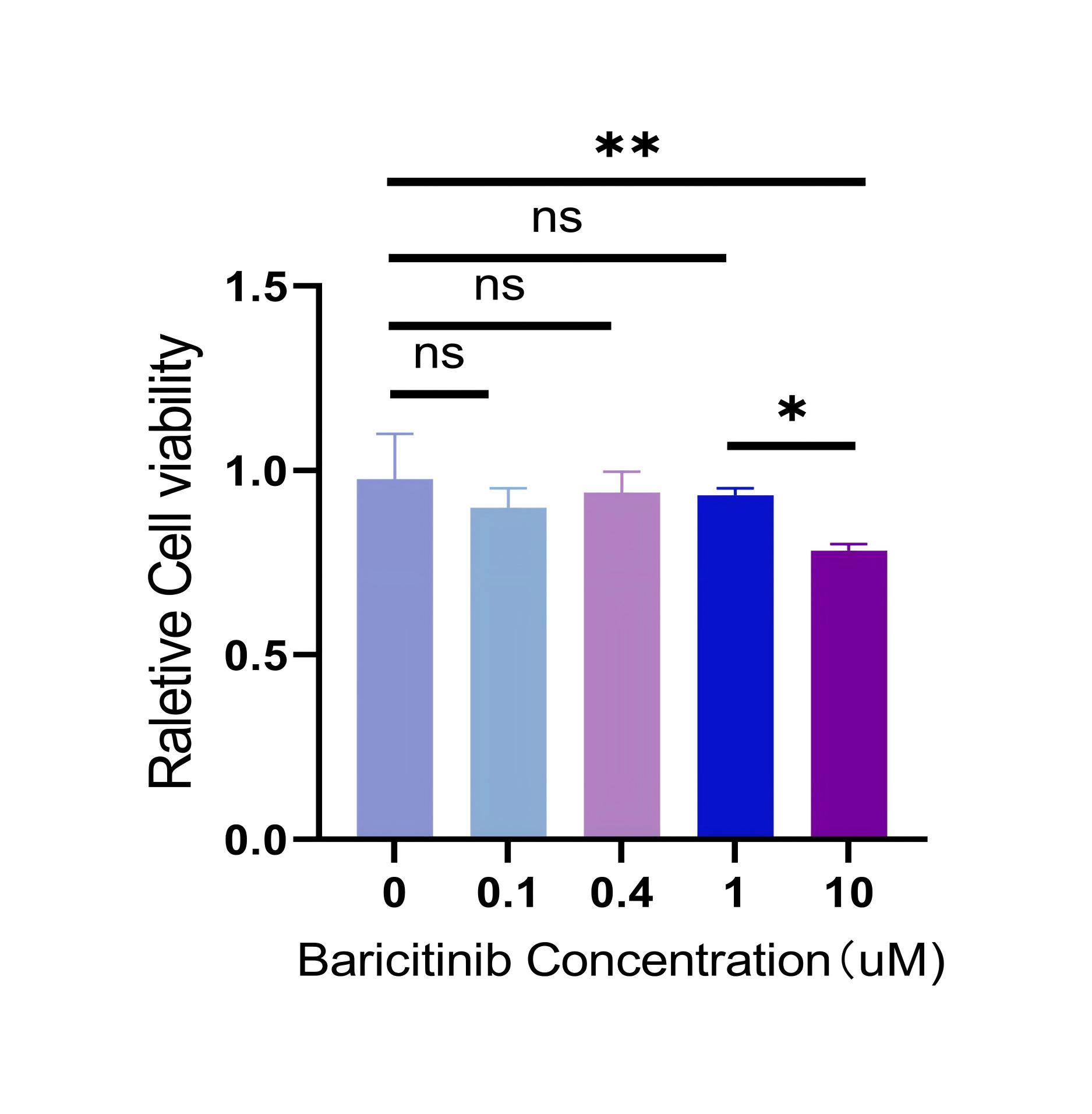

Supplement: Supplementary file 3 [file Image1.jpg]
